# Effect of exercise on nutrition, inflammation, muscle health and cardio-cerebrovascular events in maintenance hemodialysis patients: a real-world prospective cohort study

**DOI:** 10.1080/0886022X.2025.2598982

**Published:** 2025-12-18

**Authors:** Xiaoyue Yuan, Yunfeng Xia

**Affiliations:** Department of Nephrology, Metabolism and Immunology Laboratory for Urological Diseases, The First Affiliated Hospital of Chongqing Medical University, Chongqing, China

**Keywords:** Physical activity, exercise, maintenance hemodialysis, cardio-cerebrovascular events, muscle health, Malnutrition-Inflammation complex syndrome

## Abstract

Maintenance hemodialysis (MHD) patients face high risks of malnutrition-inflammation complex syndrome, muscle atrophy, and cardio-cerebrovascular events (CCVE). These challenges have led to increasing attention on the importance of exercise for MHD patients’ health. This prospective study evaluated the impact of daily physical activity (PA) on multidimensional health outcomes in this population. A total of 307 MHD patients were enrolled and divided into three groups based on their daily PA levels according to the Physical Activity Rating Scale: inactive (Group A), low PA (Group B), and moderate-to-high PA (Group C). A follow-up and comparison of differences in nutrition, inflammation, muscle health, and the occurrence of CCVE were conducted among the three groups before and after the study. At baseline, patients in group C had better malnutrition-inflammation scores, calf circumference, handgrip strength, Ishii scores, prealbumin, and albumin than the other two groups. After 11.9 ± 0.25 months of follow-up, all the aforementioned indicators deteriorated to varying degrees in Groups A and B, whereas Group C showed no significant deterioration. Furthermore, Group C showed improvements in mid-arm muscle circumference and transferrin. During the follow-up period, 52 patients experienced CCVE. Both Kaplan-Meier survival analysis and multivariate Cox regression analysis revealed that active exercise was associated with reduced risk of CCVE occurrence, particularly in patients undergoing dialysis for one to five years or those with comorbid diabetes. In conclusion, active exercise is associated with improvements in malnutrition, inflammation, and muscle health while potentially reducing the risk of CCVE in MHD patients.

## GRAPHICAL ABSTRACT

**Figure:**
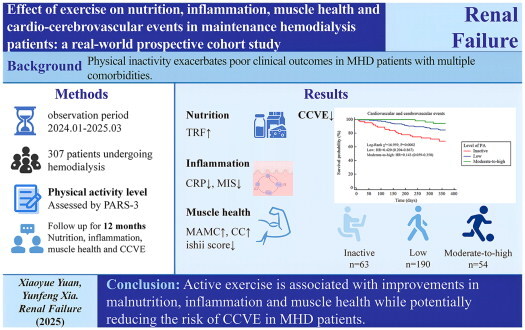

## Introduction

With the increasing number of patients with end-stage kidney disease (ESKD), studies report that approximately 3.9 million ESKD patients worldwide are currently receiving kidney replacement therapy [1]. Hemodialysis is the primary treatment for ESKD patients, but as an incomplete form of kidney replacement therapy, it cannot fully correct metabolic disturbances and toxin accumulation in ESKD patients. Furthermore, hemodialysis promotes nutrient loss and enhances catabolism, which increases the risk of malnutrition and chronic microinflammatory state in maintenance hemodialysis (MHD) patients and accelerates the onset and progression of complications such as atherosclerotic cardio-cerebrovascular disease and uremic sarcopenia, severely threatening patients’ quality of life and prognosis [2–4]. Studies report that approximately 28–54% of MHD patients are malnourished, 25.6% are comorbid with sarcopenia, and cardiovascular disease is the main cause of death in these patients [4–6]. Thus, although dialysis prolongs the survival time of MHD patients, their prognosis remains suboptimal. There is an urgent need to explore effective and comprehensive intervention strategies.

Physical activity (PA), defined as any bodily movement produced by skeletal muscle contraction resulting in energy expenditure, plays a crucial role in maintaining health and preventing disease [7]. However, inadequate physical activity is prevalent across all stages of chronic kidney disease (CKD), particularly among MHD patients, with up to 94% reported to be insufficiently active [8]. Recent research has evaluated the potential of exercise to improve clinical outcomes in MHD patients, finding that active physical exercise can ameliorate protein-energy wasting and inflammation, enhance cardiovascular function and quality of life, and reduce mortality risk [9–11]. The KDIGO 2024 Clinical Practice Guideline for the Evaluation and Management of CKD provides specific recommendations for physical activity in CKD patients [12], but most of the referenced studies are based on randomized controlled trials (RCTs), where strict inclusion criteria and intervention protocols limit the generalizability of findings to real-world settings [13]. Moreover, many studies have small sample sizes, short follow-up periods, and lack long-term observational evidence on the association between PA and cardio-cerebrovascular events (CCVE) [9]. This prospective cohort study aims to systematically evaluate the long-term effects of different PA levels on nutritional status, inflammation, muscle health, and CCVE risk in MHD patients within a real-world setting and to explore potential mechanisms, with the hope of providing a scientific reference for daily exercise regimens in this population.

## Methods

### Participants

Patients undergoing MHD from the First Affiliated Hospital of Chongqing Medical University between January 2024 and March 2025 were recruited. Inclusion criteria were as follows: (1) Age 18–75 years, undergoing hemodialysis three times a week for 3–4 h per session, and maintained for at least 3 months; (2) Single-pool Kt/*V* > 1.2 per session; (3) Relatively stable medical status without severe infection or malignancy. Exclusion criteria were as follows: (1) Combined with severe cardiopulmonary diseases, such as recurrent heart failure, severe arrhythmia, unstable angina, severe pericardial effusion, severe valvular heart disease, hypertrophic cardiomyopathy, aortic dissection, severe emphysema, pulmonary heart disease, and severe pulmonary hypertension (mean pulmonary artery pressure > 55 mmHg); (2) Combined with chronic joint, muscle, or vascular diseases precluding or unsuitable for regular exercise, such as cerebrovascular disease sequelae, severe lower limb joint impairment, and deep vein thrombosis; (3) Patients unwilling to participate in the study. This study has been registered with the Clinical Trial Center (NCT06568835) and has obtained approval from the Ethics Committee of the First Affiliated Hospital of Chongqing Medical University (permission number: 2024-034-01). The study complied with the Declaration of Helsinki, and written informed consent was obtained from all participants.

### Data collection

Clinical, laboratory, and anthropometric data were collected, including age, gender, dialysis duration, primary disease, the occurrence of intradialytic hypotension (IDH), comorbidities (including hypertension, diabetes mellitus, history of cardio-cerebrovascular disease), medication use (renin-angiotensin system inhibitors (RASIs), platelet inhibitors), smoking status, daily protein intake level (assessed by a simplified food frequency questionnaire) [14], Malnutrition-Inflammation score (MIS) [15], serum prealbumin (PAB), albumin (Alb), transferrin (TRF), hemoglobin (Hb), platelet count (PLT), C-reactive protein (CRP), blood urea nitrogen (BUN), creatinine (Cr), calcium (Ca), phosphate (P), intact parathyroid hormone (iPTH), total cholesterol (TC), high-density lipoprotein cholesterol (HDL-C), low-density lipoprotein cholesterol (LDL-C), and triglyceride (TG). Anthropometric measurements included Body Mass Index (BMI), non-fistula arm measurements (mid-arm circumference (MAC), triceps skinfold (TSF), mid-arm muscle circumference (MAMC)), calf circumference (CC), handgrip strength (HGS), and Ishii score [16].

### Assessment of physical activity level and grouping

Patients engaged in physical activity spontaneously and did not receive structured counseling. Physical activity level was assessed and grouped at baseline using the Physical Activity Rating Scale (PARS-3) revised by Liang Deqing et al. [17]. This scale comprises three items (exercise intensity, frequency, and duration), each scored on a 1–5 scale. The total PARS-3 score = intensity × frequency × (duration − 1). Scores were categorized as low PA (≤19), moderate PA (20–42), and high PA (≥43). In this study, patients were divided into three groups based on PARS-3 score: Inactive Group (Group A, PARS-3 score ≤4), Low PA Group (Group B, PARS-3 score 5–19), and Moderate-to-High PA Group (Group C, PARS-3 score ≥20) [18,19]. The test-retest reliability of PARS-3 was 0.82.

### Patients follow-up

Patients were followed for approximately 12 months. Changes in clinical, laboratory, and anthropometric indicators, PA level, and occurrences of CCVE were closely monitored and recorded. CCVE included acute heart failure, symptomatic arrhythmia episodes, acute coronary syndrome, cerebral hemorrhage, ischemic stroke, and transient ischemic attack. The primary endpoint was death or occurrence of CCVE. The secondary endpoint was hospitalization for any cause.

### Statistical analyses

The statistical analyses were performed using SPSS 26.0 software and R 4.3.2. Normally distributed continuous data are presented as mean ± standard deviation (SD), and group comparisons were made using analysis of variance (ANOVA). Non-normally distributed continuous data are presented as median (interquartile range, IQR), and groups comparisons used the Kruskal-Wallis test. Categorical data are presented as counts and percentages, and group comparisons used the chi-test or Fisher’s exact test. The Bonferroni *post hoc* analysis was performed to determine statistically significant changes after one-way ANOVA. Analysis of covariance (ANCOVA), adjusting for potential confounders and baseline values, was used to analyze the impact of PA level on nutritional, inflammatory, muscle mass, and strength indicators. Paired sample *t*-tests were used for within-group comparisons of indicators before and after the study. Kaplan–Meier survival curves with log-rank tests were used to compare differences in CCVE incidence and hospitalization rates between different PA groups; pairwise comparisons between groups were adjusted for *P*-values using the Bonferroni method. Multivariable Cox proportional hazards models were constructed using a Change-in-Estimate (CIE) approach to select covariates. Demographic characteristics and variables with a *p*-value < 0.10 in univariate analyses were considered. Those not significantly associated with CCVE were removed from the final model, unless doing so altered the physical activity hazard ratio (HR) by ≥10%. Additionally, the dose-response relationship between the continuous PARS-3 score and CCVE risk was assessed using restricted cubic splines. Subgroup analyses were performed based on age, gender, dialysis duration, diabetes, cardio-cerebrovascular disease history, and nutritional status, calculating HR with 95% confidence intervals (95% CI) for CCVE in each subgroup. Interaction effects were tested using likelihood ratio tests. Two-sided *p*-values <0.05 were considered statistically significant.

## Results

### Comparison of baseline characteristics among the three groups

A total of 314 eligible MHD patients were recruited. During follow-up, two patients underwent kidney transplantation, and five patients were transferred to other dialysis centers. Ultimately, 307 patients were included in the statistical analysis. Based on the baseline PARS-3 score, 63 patients were assigned to Group A, 190 to Group B, and 54 to Group C (Supplementary Figure S1). In this cohort, the vast majority of patients (*n* = 292, 95.1%) engaged primarily in aerobic activities (such as walking, jogging, or Tai Chi). A very small proportion of patients reported resistance training (*n* = 2, 0.7%) or a mixed regimen (*n* = 2, 0.7%) as their main form of exercise. Additionally, 11 patients (3.6%) did not engage in any exercise (PARS-3 score = 0).

Comparison of baseline characteristics revealed that patients in the lower PA groups (A and B) were relatively older, had longer dialysis duration, poorer nutritional status, and lower daily protein intake level. Group C patients had significantly higher CC, HGS, PAB, Alb, BUN, Cr, and HDL-C levels compared to Groups A and B, and significantly lower Ishii scores. No significant differences were found for other indicators among the three groups (Table 1).

**Table 1.** Baseline characteristics of study patients according to the physical activity levels.

Characteristics	Inactive (*n* = 63)	Low (*n* = 190)	Moderate-to-high (*n* = 54)	*F/H/χ* ^2^	*P value*
Clinical data					
Sex, n (%) Female Male	32 (50.8) 31 (49.2)	98 (51.6) 92 (48.4)	21 (38.9) 33 (61.1)	2.791	0.248
Age (yrs)	56.35 ± 13.08	54.43 ± 11.57	50.87 ± 11.46^a^	3.192	0.042
Dialysis duration (mo)	96.43 ± 66.65	79.84 ± 56.57	62.70 ± 46.27^a^	5.071	0.007
Smoking status, n (%) Never Former Current	32 (50.8) 17 (27.0) 14 (22.2)	117 (61.6) 35 (18.4) 38 (20.0)	27 (50.0) 12 (22.2) 15 (27.8)	4.469	0.346
Primary disease, n (%)				4.414	0.621
Chronic nephritis	27 (42.9)	102 (53.7)	31 (57.4)		
Diabetic nephropathy	14 (22.2)	41 (21.6)	8 (14.8)		
Hypertensive	7 (11.1)	17 (8.9)	5 (9.3)		
Others	15 (23.8)	30 (15.8)	10 (18.5)		
IDH, n (%)	20 (31.7)	35 (18.4)	11 (20.4)	5.027	0.081
With hypertension, n (%)	62 (98.4)	186 (97.9)	52 (96.3)	0.653	0.721
With diabetes mellitus, n (%)	19 (30.2)	52 (27.4)	10 (18.5)	2.277	0.320
With CCVD, n (%)	16 (25.4)	36 (18.9)	5 (9.3)	5.056	0.080
MIS score	7.44 (3.01)	5.45 (2.53) ^a^	4.56 (2.34) ^ab^	20.261	<0.001
Nutritional status Well-nourished, n (%) Malnourished, n (%)	19 (30.2) 44 (69. 8)	109 (56.3) 83 (43.7) ^a^	40 (74.1) 14 (25.9) ^b^	23.591	<0.001
RASIs therapy, n (%)	29 (46.0)	93 (48.9)	24 (44.4)	0.416	0.812
Antiplatelets user, n (%)	33 (52.4)	77 (40.5)	18 (33.3)	4.619	0.099
Day protein intake (g/(kg·d))	0.67 ± 0.18	0.76 ± 0.19^a^	0.83 ± 0.19^a^^b^	11.742	<0.001
Anthropometric data					
BMI (Kg/m^2^)	22.32 ± 4.06	22.87 ± 3.42	22.44 ± 3.21	0.716	0.489
MAC (cm)	25.19 ± 3.41	26.22 ± 2.82	26.06 ± 2.63	2.934	0.055
TSF (cm)	1.41 ± 0.84	1.46 ± 0.66	1.43 ± 0.64	0.136	0.873
MAMC (cm)	20.76 ± 2.53	21.63 ± 2.50	21.58 ± 2.40	3.010	0.051
CC (cm)	32.01 ± 3.24	32.96 ± 2.88	33.64 ± 2.52^a^	4.793	0.009
HGS (kg)	14.85 ± 5.53	17.62 ± 6.89^a^	21.28 ± 6.25^a^^b^	14.142	<0.001
Weighted HGS (kg/kg)	0.25 ± 0.078	0.29 ± 0.096^a^	0.35 ± 0.100^a^^b^	17.748	<0.001
Ishii score	139.16 ± 28.78	124.21 ± 29.62^a^	107.81 ± 26.15^a^^b^	17.164	<0.001
Laboratory parameters					
PAB (g/L)	283.99 ± 68.71	292.87 ± 58.85	312.55 ± 62.32^a^	3.303	0.038
Alb (g/L)	39.98 ± 3.92	40.88 ± 3.36	41.70 ± 3.55^a^	3.520	0.031
TRF (g/L)	2.17 ± 0.50	2.15 ± 0.42	2.12 ± 0.37	0.202	0.818
CRP (mg/L)	3.99 (7.34)	3.48 (4.38)	2.88 (4.63)	3.000	0.223
BUN (mmol/L)	20.24 ± 5.86	22.65 ± 5.96^a^	22.39 ± 5.67	4.030	0.019
Cr (μmol/L)	846.84 ± 231.99	924.81 ± 208.71^a^	940.26 ± 235.28	3.581	0.029
Ca (mmol/L)	2.25 ± 0.22	2.25 ± 0.20	2.22 ± 0.21	0.433	0.649
P (mmol/L)	1.73 ± 0.44	1.74 ± 0.69	1.86 ± 0.58	0.854	0.427
iPTH (pg/mL)	265.70 (355.70)	253.35 (378.40)	322.20 (399.33)	0.538	0.764
TC (mmol/L)	3.81 ± 1.16	3.87 ± 0.80	4.00 ± 0.91	0.592	0.555
TG (mmol/L)	2.38 ± 2.44	2.05 ± 1.18	2.00 ± 1.42	0.608	0.546
HDL-C (mmol/L)	1.00 ± 0.29	1.07 ± 0.33	1.18 ± 0.41^a^	4.034	0.019
LDL-C (mmol/L)	2.12 ± 0.88	2.12 ± 0.67	2.19 ± 0.72	0.230	0.795
Hb (g/L)	113.32 ± 16.29	115.33 ± 12.94	114.12 ± 13.58	0.559	0.572
PLT (10^9^/L)	184.33 ± 58.17	186.01 ± 60.64	187.02 ± 56.78	0.032	0.969

Note: Data was presented as mean ± SD or median (IQR) or number (percentage). hysical activity level: inactive, PARS-3 ≤ 4; low, 4 < PARS-3 < 20; moderate-to-high, PARS-3 ≥ 20. Nutritional status: well-Nourished, MIS ≤ 5; Malnourished, MIS ≥6.

^a^ Significant versus Inactive.

^b^ Significant versus Low.

Abbreviation: SD: standard deviation; IQR: interquartile range; CCVD: cardio-cerebrovascular disease; MIS score: malnutrition inflammation score; RASIs: renin-angiotensin system inhibitors; BMI: body mass index; MAC: mid-arm circumference; TSF: triceps skinfold thickness; MAMC: mid-arm muscle circumference; CC: calf circumference: HGS: handgrip strength; PAB: prealbumin; Alb: albumin; TRF: transferrin; CRP: C-reactive protein; BUN: blood urea nitrogen; Cr: creatinine; Ca: calcium; P: phosphate; iPTH: intact parathyroid hormone; TC: total cholesterol; TG: triglyceride; HDL-C: high-density lipoprotein cholesterol; LDL-C: low-density lipoprotein cholesterol; Hb: hemoglobin; PLT: Platelet. IDH: Intradialysis hypotension, generally denotes a decrease in systolic blood pressure of ≥20 mmHg or a reduction in mean arterial pressure of ≥10 mmHg during hemodialysis, accompanied by symptoms of hypotension.

### Effect of daily physical activity level on nutrition, inflammation, muscle mass, and strength in MHD patients

After 11.9 ± 0.25 months of follow-up, ANCOVA analysis adjusted for baseline values and confounders revealed that patients in Groups B and C had significantly better MAMC, CC, Ishii score, and MIS score compared to Group A. There were no significant differences in MAC, HGS, PAB, Alb, TRF, CRP, and serum creatinine among the three groups (Supplementary Table S1).

Within-group before-and-after comparisons showed that Group A patients experienced significant worsening in MAC, CC, Ishii score, MIS score, PAB, Alb, and CRP. Group B patients also showed significant worsening in MAC, PAB, and Alb. Only Group C patients showed no significant deterioration in any measured indicators and significant improvement in MAMC and serum TRF (Table 2).

**Table 2.** Longitudinal comparison of physiological indicators at baseline and endpoint of the follow-up by physical activity levels.

	Inactive (*n* = 63)				Low (*n* = 190)				Moderate-to-high (*n* = 54)			
Characteristics	Baseline	12 months	*T/Z*	*P*	Baseline	12 months	*T/Z*	*P*	Baseline	12 months	*T/Z*	*P*
MAC (cm)	25.19 (3.41)	24.86 (3.26)	3.30	0.002	26.22 (2.82)	25.99 (2.96)	2.91	0.004	26.06 (2.63)	25.93 (2.38)	0.78	0.442
MAMC (cm)	20.76 (2.53)	20.59 (2.55)	1.62	0.110	21.63 (2.50)	21.71 (2.63)	−1.07	0.288	21.58 (2.40)	21.98 (2.47)	−2.05	0.045
CC (cm)	32.01 (3.24)	31.56 (3.17)	5.27	<0.001	32.96 (2.88)	32.83 (2.87)	1.81	0.071	33.64 (2.52)	33.80 (2.46)	−1.54	0.129
HGS (kg)	14.85 (5.53)	14.41 (5.78)	1.82	0.074	17.62 (6.89)	17.52 (6.96)	0.84	0.400	21.28 (6.25)	21.07 (6.52)	0.65	0.520
Weighted HGS (kg/kg)	0.25 (0.078)	0.24 (0.083)	1.91	0.061	0.29 (0.096)	0.29 (0.096)	1.68	0.094	0.35 (0.100)	0.35 (0.103)	0.60	0.551
Ishii score	139.16 (28.78)	142.89 (29.93)	−3.38	0.001	124.21 (29.62)	125.05 (30.52)	−1.37	0.174	107.81 (26.15)	106.72 (25.37)	0.96	0.342
MIS score	7.44 (3.01)	8.10 (3.30)	−2.50	0.015	5.45 (2.53)	5.66 (2.85)	−1.63	0.104	4.56 (2.34)	4.37 (2.18)	0.94	0.349
PAB (g/L)	283.99 (68.71)	264.54 (69.68)	2.93	0.005	292.87 (58.85)	285.11 (56.69)	2.30	0.023	312.55 (62.32)	306.85 (55.87)	0.78	0.437
Alb (g/L)	39.98 (3.92)	38.38 (4.53)	3.36	0.001	40.88 (3.36)	39.79 (3.54)	4.20	<0.001	41.70 (3.55)	40.96 (3.03)	1.73	0.089
TRF (g/L)	2.17 (0.50)	2.17 (0.43)	0.04	0.972	2.15 (0.42)	2.19 (0.41)	−1.51	0.132	2.12 (0.37)	2.26 (0.51)	−2.42	0.019
LnCRP (mg/L)	1.54 (0.95)	1.80 (1.02)	−2.86	0.006	1.39 (0.86)	1.47 (0.93)	−1.48	0.141	1.22 (0.95)	1.47 (0.90)	−1.99	0.052
Cr (μmol/L)	846.84 (231.99)	871.30 (243.97)	−1.20	0.235	924.81 (208.71)	945.45 (234.83)	−1.78	0.077	940.26 (235.28)	978.48 (208.94)	−1.99	0.052

Note: Data was presented as mean (SD).

Abbreviation: SD: standard deviation; MAC: mid-arm circumference; MAMC: mid-arm muscle circumference; CC: calf circumference: HGS: handgrip strength; MIS score: malnutrition inflammation score; PAB: prealbumin; Alb: albumin; TRF: transferrin; LnCRP: natural logarithm of C-reactive protein; Cr: creatinine.

### Effect of daily physical activity level on cardio-cerebrovascular events in MHD patients

**Figure 1.**
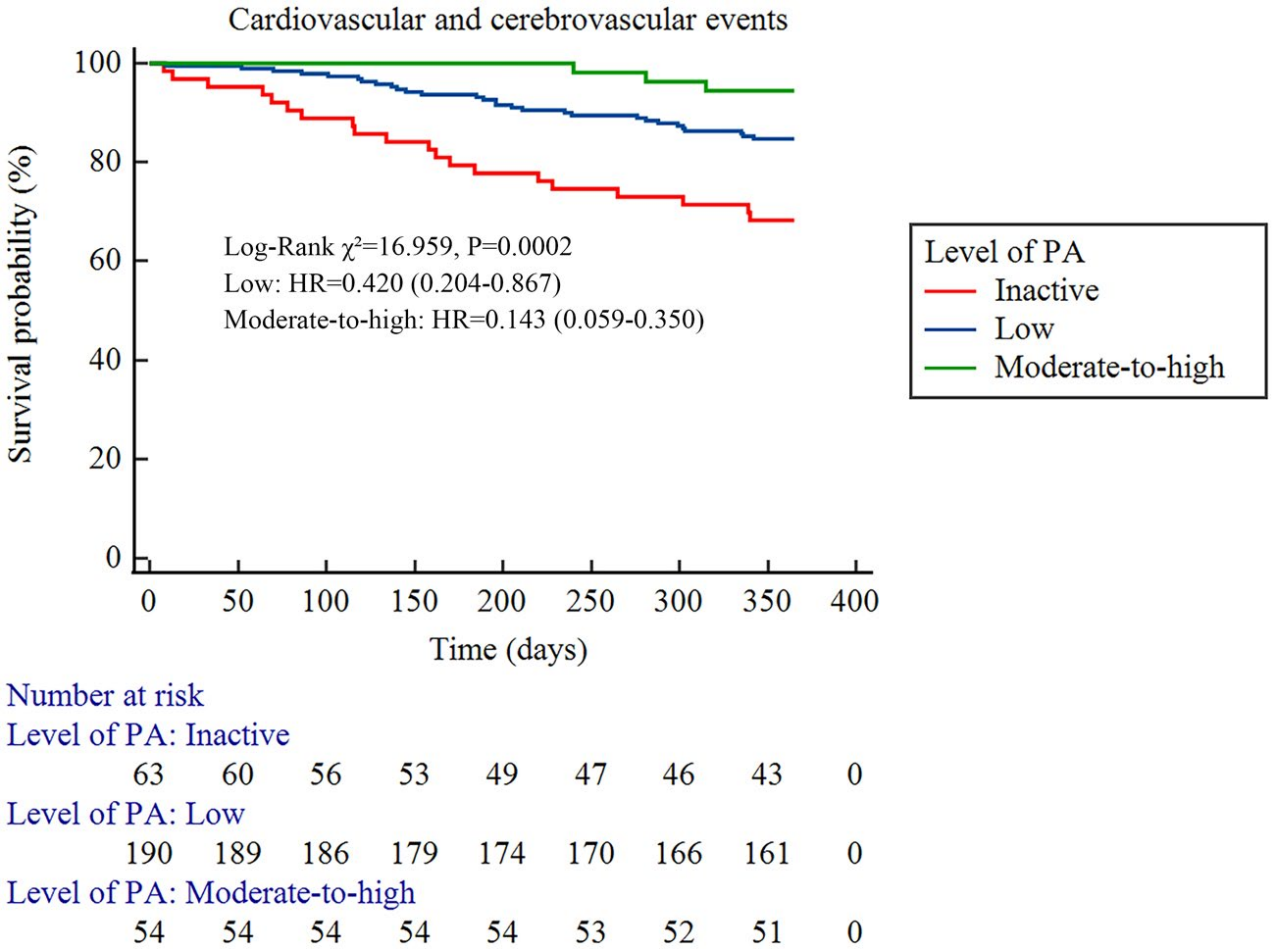
Survival analysis of different physical activity levels and the occurrence of CCVE in MHD patients (Kaplan-Meier Survival Curve).

**Figure 2.**
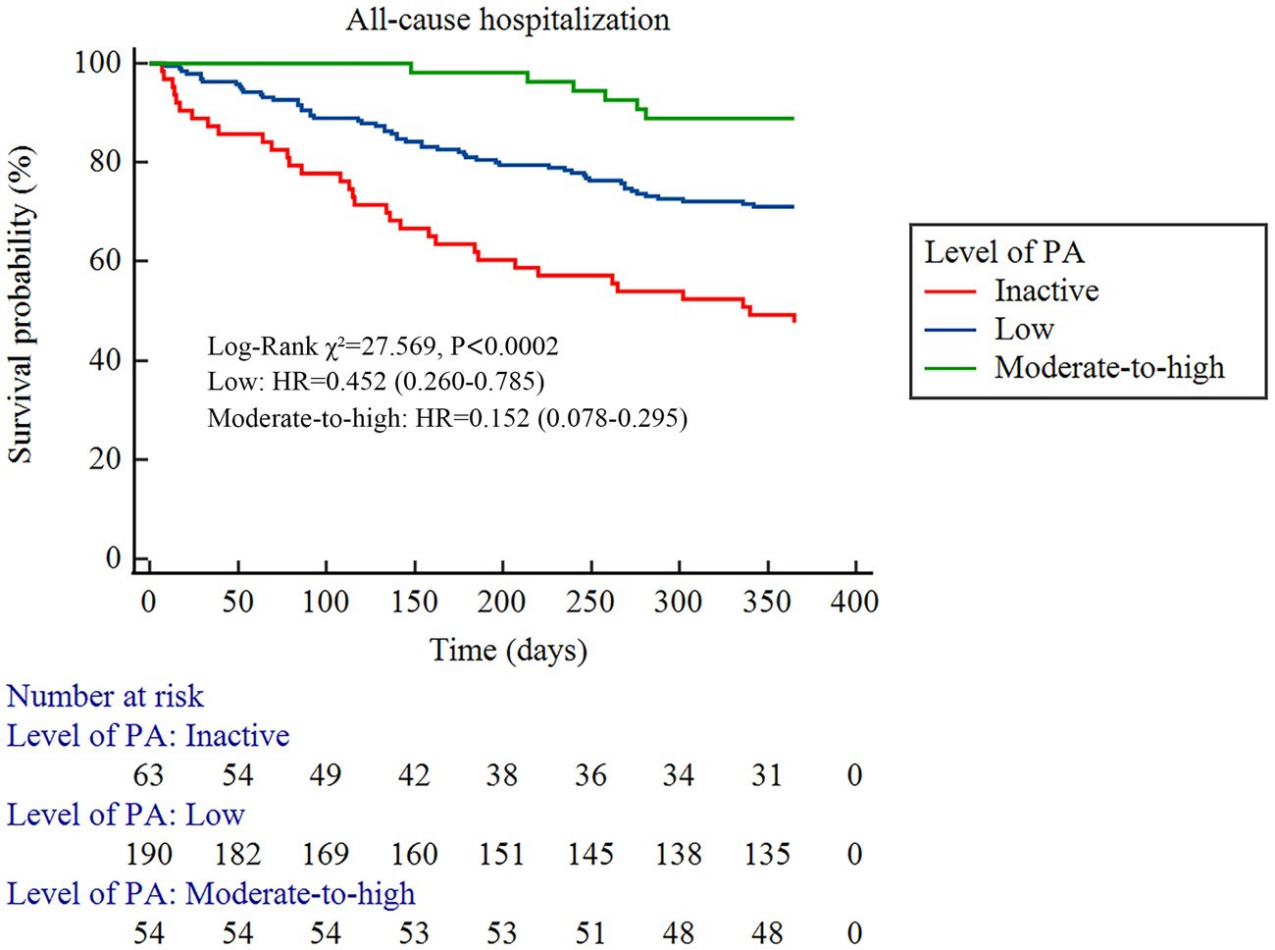
Survival analysis of different physical activity levels and the occurrence of hospitalizations in MHD patients (Kaplan-Meier Survival Curve).

Fifty two patients experienced CCVE during follow-up, including 22 acute coronary syndromes, 12 acute heart failures, 12 ischemic strokes, and 6 cerebral hemorrhages. Three patients died due to CCVE, two from Group A and one from Group B. The incidence rates of CCVE in Groups A, B, and C were 31.75% (20/63), 15.26% (29/190), and 5.56% (3/54), respectively. Kaplan-Meier survival analysis showed a significant difference in the cumulative incidence of CCVE among the three groups (log-rank *χ*^2^=16.959, *p* = 0.0002) (Figure 1). Post-hoc pairwise comparisons with Bonferroni correction revealed that both Group B (*p* = 0.002) and Group C (*p* < 0.001) had a significantly lower risk than Group A, although no significant difference was observed between Group B and C (*p* = 0.062). During follow-up, 94 patients required hospitalization, primarily for CCVE (*n* = 52, 55.3%), followed by vascular access-related complications (*n* = 25, 26.6%), infections (*n* = 13, 13.8%), and other causes (*n* = 4, 4.3%). Kaplan-Meier survival analysis also showed a significant difference in cumulative hospitalization incidence (log-rank *χ*^2^=27.569, *p* < 0.0001) (Figure 2). All pairwise comparisons between groups remained statistically significant (*p* < 0.01).

Univariate Cox regression analysis revealed that age, history of diabetes, history of cardio-cerebrovascular disease (CCVD), malnutrition, use of RASIs, HGS, Ishii score, PAB, Alb, TC, and PA level were significantly associated with CCVE risk in MHD patients (Supplementary Table S2). The final multivariable Cox model was constructed using the CIE approach (Supplementary Table S3). After adjustment for age, PAB, TC, and LDL-C, higher PA levels remained independently associated with a significantly lower risk of CCVE. Compared to Group A, the risk of CCVE was reduced by 53.6% in Group B (HR = 0.464, 95%CI 0.260–0.826) and by 77.8% in Group C (HR = 0.222, 95%CI 0.065–0.759) (Table 3).

**Table 3.** Multivariable Cox model predicting CCVE in MHD patients.

Characteristics	HR (95%CI)	*P*
PA level		
Inactive	1.000 (Reference)	
Low	0.464 (0.260 – 0.826)	0.009
Moderate-to-high	0.222 (0.065 – 0.759)	0.016
Age	1.049 (1.021 – 1.077)	0.001
PAB	0.994 (0.990 – 0.999)	0.013
TC	0.453 (0.231 – 0.889)	0.021
LDL-C	2.016 (0.869 – 4.678)	0.103

Note: physical activity level: inactive, PARS-3 ≤ 4; low, 4 < PARS-3 < 20; moderate-to-high, PARS-3 ≥ 20.

Abbreviation: PAB: prealbumin; TC: total cholesterol; LDL: low-density lipoprotein cholesterol.

**Figure 3.**
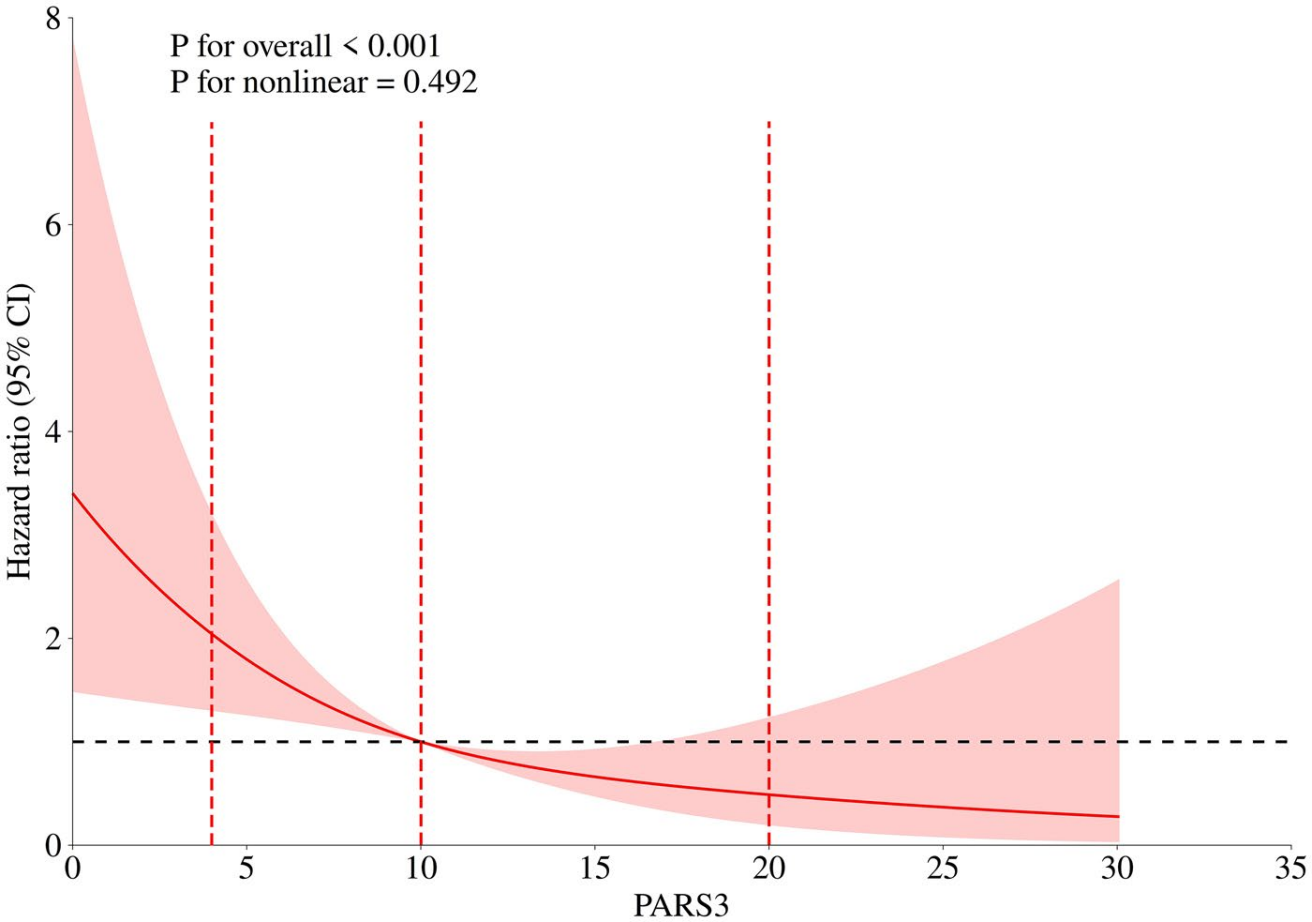
Restricted cubic spline analysis of the relationship between PARS-3 and the risk of CCVE. Note: The model was adjusted for age, PAB, TC and LDL-C. The analysis used three knots placed at the 10th, 50th and 90th percentiles of the PARS-3 score distribution, which corresponded to scores of 4, 10 and 20.

A sensitivity analysis using restricted cubic splines confirmed a significant linear inverse dose-response relationship between the continuous PARS-3 score and CCVE risk (*P* for non-linear = 0.492) (Figure 3).

### Subgroup analysis and interaction effects in MHD patients

**Figure 4.**
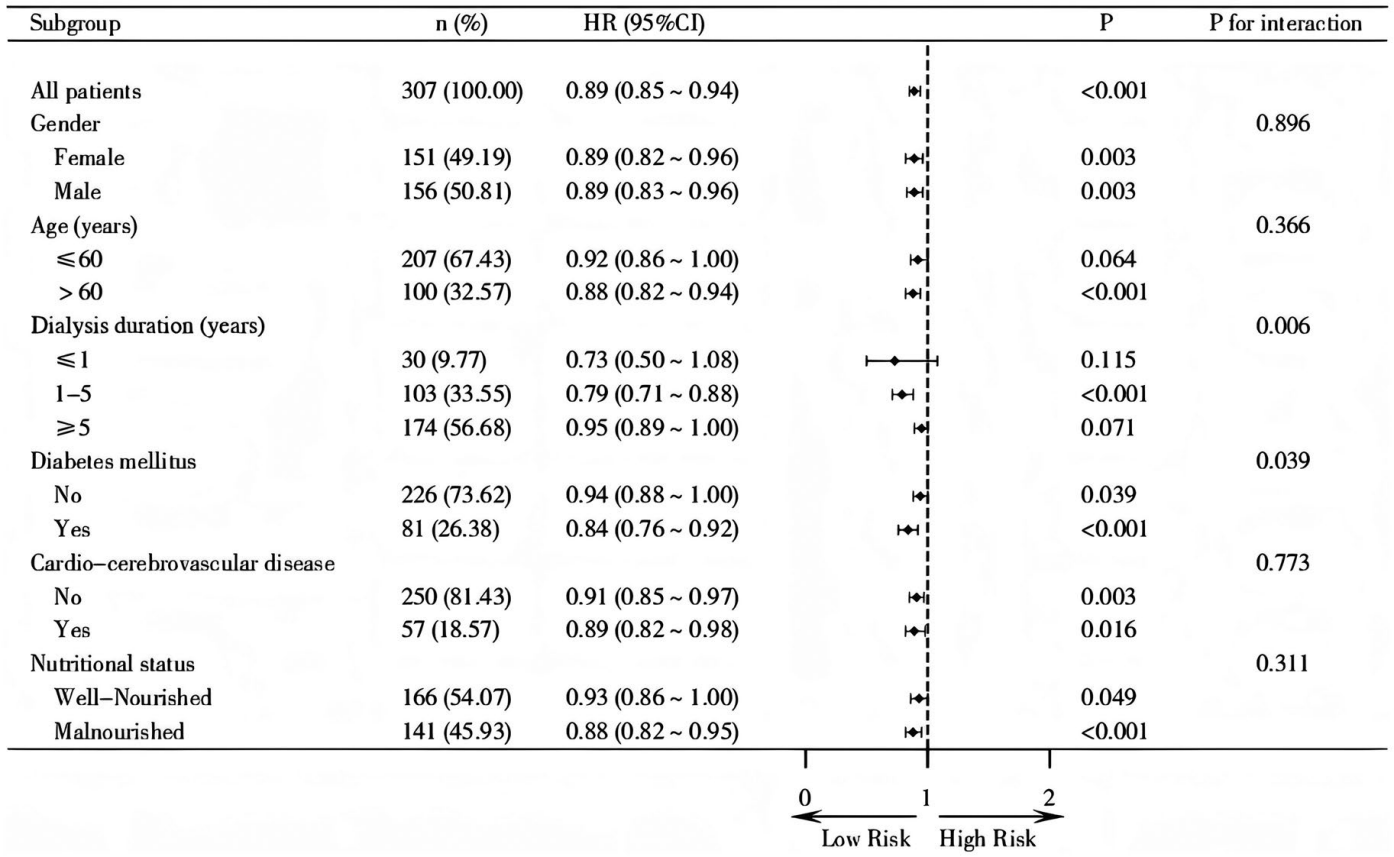
Subgroups analysis for the associations between physical activity level and CCVE in MHD patients. **Note:** Nutritional status: well-Nourished, MIS ≤ 5; Malnourished, MIS ≥6.

There was no interaction in subgroups stratified by age, gender, cardiovascular disease history, and nutritional status (*P* for interaction >0.05). However, significant interactions (*P* for interaction <0.05) were observed between dialysis duration, diabetes history, and PARS-3 score. In patients on dialysis for one to five years or with comorbid diabetes, higher levels of PA are associated with a reduced risk of CCVE (Figure 4).

## Discussions

MHD patients frequently experience malnutrition and a state of chronic microinflammation, which are interrelated and often referred to as the malnutrition-inflammation complex syndrome (MICS) [20]. MICS can induce endothelial cell dysfunction, promote oxidative stress damage, and facilitate atherosclerosis. Notably, atherosclerosis is essentially an immune-inflammatory disease affecting medium and large arteries, closely associated with life-threatening clinical events such as acute coronary syndrome and stroke [21]. Furthermore, MICS inhibits muscle protein synthesis while promoting its degradation, ultimately leading to muscle atrophy and functional decline. This represents one of the key mechanisms underlying the development of sarcopenia and CCVE in MHD patients [22–24]. Consequently, the assessment and management of MICS are crucial in clinical practice. Nutritional markers such as Alb, PAB, and TRF are not only objective indicators for assessing protein-energy wasting and nutritional status but are also closely associated with the state of microinflammation [25]. Regular monitoring of these nutritional biomarkers helps evaluate the patient’s nutritional-inflammatory status and guides the formulation of clinical nutritional intervention plans.

In recent years, non-pharmacological interventions aimed at improving patient prognosis by increasing PA levels have gained increasing attention. However, due to factors like renal failure, post-dialysis fatigue, and a heavy burden of comorbidities, most MHD patients significantly reduce their daily physical activity [8]. Prolonged low levels of PA further exacerbate adverse health outcomes, including cardiovascular disease, hospitalization, and death [26,27]. In this prospective cohort study, we found that approximately 20% of MHD patients lacked basic physical exercise over the long term. After nearly 12 months of follow-up, patients in the inactive and low PA groups showed varying degrees of deterioration in MICS-related indicators (such as the MIS, Alb, PAB, and TRF), muscle mass, and strength indicators. In contrast, patients maintaining moderate to high levels of PA exhibited no significant deterioration in these indicators; they even demonstrated improvements in MAMC and TRF. These results further confirm that regular exercise may ameliorate malnutrition, inflammatory status, and muscle mass in MHD patients to some extent, consistent with findings from previous studies [28–30].

Handgrip strength is an important indicator for assessing upper limb muscle strength and indirectly reflecting nutritional status. In this study, no significant changes were observed in patients’ handgrip strength and serum albumin levels with increasing levels of PA. This finding may be attributed to MHD patients primarily engaging in aerobic exercises such as walking, jogging, and tai chi, coupled with protein intakes generally below the recommended dosage of 1.0–1.2g/kg/d [31]. Previous studies have shown that resistance training combined with protein supplementation can stimulate muscle protein synthesis, increase muscle mass, and improve muscle strength [32]. Therefore, combining dietary interventions with exercise may enhance the effect in future studies. CRP is a marker of chronic inflammation in MHD patients, and a meta-analysis based on home exercise indicated that exercise did not significantly contribute to reductions in CRP levels [33], which is also consistent with our results.

This study also found a significant negative correlation between daily PA level and CCVE risk in MHD patients. Compared to inactive patients, those in the low and moderate-to-high PA groups experienced a reduction in CCVE risk of a 53.6 and 80.3%, respectively. This indicates that regular PA not only improves physical function but also has a profoundly positive effect on improving prognosis [10,34]. Previous studies have shown that being physically active can reduce the risk of atherosclerosis and related CCVD in MHD patients by improving malnutrition and inflammatory status. Additionally, regular exercise can improve traditional cardiovascular risk factors like weight, blood pressure, lipids, and glucose [35]. Furthermore, aerobic exercise that engages large muscle groups, particularly the lower limbs, can enhance shear stress on blood vessels, stimulating increased nitric oxide release and bioavailability, while strengthening vagal activity and reducing sympathetic activity and vasoconstrictor strength at rest, thereby enhancing vasodilation and improving vascular function [10,36,37]. Regular moderate-intensity aerobic exercise also regulates the body’s antioxidant defense mechanisms, reduces the generation and release of reactive oxygen species, and alleviates oxidative damage to the vascular endothelium [11,37].

It is particularly noteworthy that our subgroup analysis results suggest that increasing PA level may have an even more significant effect on reducing CCVE risk among MHD patients with comorbid diabetes. This finding is reported for the first time in relevant studies and may be attributed to the combined effect of aerobic and resistance exercise in enhancing the activity of glucose transport proteins in skeletal muscle cells, promoting glucose uptake by muscle, and improving insulin resistance [38]. Additionally, studies have shown that exercise can reduce the accumulation of advanced glycation end products and mitigate the lipid and glucose toxicity on the vascular endothelium, thereby delaying the atherosclerotic process associated with diabetes [39]. Therefore, developing more aggressive exercise regimens for the diabetic population among MHD patients may be highly necessary. The study also found that the cardiovascular protective effects of exercise were more prominent in patients with a dialysis duration of one to five years. This period represents the highest risk of mortality for ESKD patients, with approximately 60% of MHD patients dying during this timeframe, primarily due to CCVE, according to data from the United States Renal Data System [40]. Given the physiological instability in the early dialysis phase (<1 year), as well as the significantly worsened vascular pathology and potential survival bias in the late period (>5 years) that may limit the benefits of exercise [41], the period of the one to five year dialysis window represents a crucial period for CCVE prevention and intervention.

MHD patients represent a high-risk population, where the long-term disease burden and dependence on hemodialysis lead to psychological distress, including depression and other psychological disorders [42], significantly limiting the participation of these patients in physical activity-related RCTs. And many intervention protocols in RCTs are often too stringent to be widely implemented in real-world settings. As a real-world prospective cohort study exploring the impact of daily PA levels in MHD patients, our work provides supportive evidence from a clinical practice setting for the PA recommendations in the KDIGO 2024 CKD Guideline [12].

This study has several limitations. First, it is a single-center observational study with a relatively small sample size. Although we adjusted for several known confounders in our statistical analysis, potential confounders such as total calorie and micronutrient intake remain, which may confound the association between physical activity and the outcome. Furthermore, the observational design cannot fully exclude the possibility of reverse causation, whereby patients with better health status may be more likely to exercise, rather than exercise alone accounting for all the benefits observed. Second, the assessment of PA relied on the PARS-3 scale, which is susceptible to recall bias and lacks objective measurements. Finally, the 12-month follow-up period remains relatively short for comprehensively evaluating the long-term impact of exercise on patient prognosis. Future studies should adopt multicenter designs with extended follow-up, incorporating objective PA monitoring (e.g., accelerometers), stratification by exercise modality, and RCTs that combine physical activity with nutritional interventions.

In conclusion, this study investigated the effects and potential mechanisms of PA on nutrition, inflammation, muscle health, and the risk of CCVE in MHD patients. The results indicated that higher levels of daily PA are associated with improvements in malnutrition, reductions in inflammation, enhancements in muscle health, and a lower risk of CCVE in MHD patients. To our knowledge, this is the first prospective, real-world study showing self-directed PA association with CCVE risk reduction in MHD patients. Furthermore, our study provides novel and unique insights by identifying that patients with diabetes and those within a one to five year dialysis window may derive the greatest cardiovascular benefit from an active lifestyle, highlighting key target populations for future interventions.

## Supplementary Material

Supplementary_Table_S2.docx

Supplementary Figure S1.tif

Supplementary Table S1.docx

Supplementary Table S3.docx

## Data Availability

The data underlying this article will be shared upon reasonable request from the corresponding author.
