# Supplementary material for: Effect of exercise on nutrition, inflammation, muscle health and cardio-cerebrovascular events in maintenance hemodialysis patients: a real-world prospective cohort study
Source: Ren Fail. 2025 Dec 18;47(1):2598982. doi: 10.1080/0886022X.2025.2598982 (PMC12720627; doi:10.1080/0886022X.2025.2598982)
Supplement: Supplementary_Table_S2.docx [file IRNF_A_2598982_SM9363.docx]

**Supplementary Table S2.** Univariate Cox regression predicting CCVE in MHD patients.

| Characteristics | HR (95%CI) | *P* |
| --- | --- | --- |
| Male | 1.048 (0.608 ~ 1.805) | 0.867 |
| Age | 1.058 (1.030 ~ 1.087) | <0.001 |
| Dialysis duration | 1.001 (0.996 ~ 1.006) | 0.638 |
| BMI | 1.011 (0.936 ~ 1.092) | 0.784 |
| Smoking | 0.713 (0.348 ~ 1.463) | 0.357 |
| Intra-dialysis hypotension | 0.867 (0.435 ~ 1.728) | 0.867 |
| With diabetes mellitus | 2.986 (1.732 ~ 5.146) | <0.001 |
| With CCVD | 3.139 (1.795 ~ 5.491) | <0.001 |
| Malnourished | 2.230 (1.268 ~ 3.291) | 0.005 |
| RASIs user | 2.074 (1.179 ~ 3.648) | 0.011 |
| Antiplatelets user | 1.694 (0.982 ~ 2.923) | 0.058 |
| PA level |  |  |
| Inactive | 1.000 (Reference) |  |
| Low | 0.418 (0.236 ~ 0.738) | 0.003 |
| Moderate-to-high | 0.143 (0.042 ~ 0.481) | 0.002 |
| MAC | 0.953 (0.0868 ~ 1.047) | 0.318 |
| TSF | 0.851 (0.563 ~ 1.286) | 0.443 |
| MAMC | 0.973 (0.872 ~ 1.085) | 0.619 |
| CC | 0.923 (0.838 ~ 1.017) | 0.106 |
| HGS | 0.939 (0.899 ~ 0.981) | 0.005 |
| Weighted HGS | 0.009 (0.000 ~ 0.182) | 0.002 |
| Ishii score | 1.020 (1.011 ~ 1.029) | <0.001 |
| PAB | 0.992 (0.988 ~ 0.996) | <0.001 |
| Alb | 0.893 (0.830 ~ 0.961) | 0.003 |
| TRF | 0.670 (0.347 ~ 1.294) | 0.233 |
| CRP | 1.018 (0.999 ~ 1.037) | 0.058 |
| TC | 0.653 (0.470 ~ 0.908) | 0.011 |
| TG | 0.865 (0.679 ~ 1.103) | 0.242 |
| HDL-C | 0.831 (0.368 ~ 1.878) | 0.657 |
| LDL-C | 0.707 (0.470 ~ 1.062) | 0.095 |
| Hb | 1.003 (0.983 ~ 1.023) | 0.793 |
| PLT | 1.001 (0.996 ~ 1.005) | 0.814 |

**Note:** physical activity level: inactive, PARS-3≤ 4; low, 4＜PARS-3＜20; moderate-to-high, PARS-3 ≥ 20.

**Abbreviation:** BMI: body mass index; CCVD: cardio-cerebrovascular disease; RASIs: renin-angiotensin system inhibitors; PA level: physical activity level; MAC: mid-arm circumference; TSF: triceps skinfold thickness; MAMC: mid-arm muscle circumference; CC: calf circumference: HGS: handgrip strength; PAB: prealbumin; Alb: albumin; TRF: transferrin; CRP: C-reactive protein; TC: total cholesterol; TG: triglyceride; HDL-C: high-density lipoprotein cholesterol; LDL-C: low-density lipoprotein cholesterol; Hb: hemoglobin; PLT: Platelet.
