# Supplementary figures and images for: Effect of exercise on nutrition, inflammation, muscle health and cardio-cerebrovascular events in maintenance hemodialysis patients: a real-world prospective cohort study
Source: Ren Fail. 2025 Dec 18;47(1):2598982. doi: 10.1080/0886022X.2025.2598982 (PMC12720627; doi:10.1080/0886022X.2025.2598982)

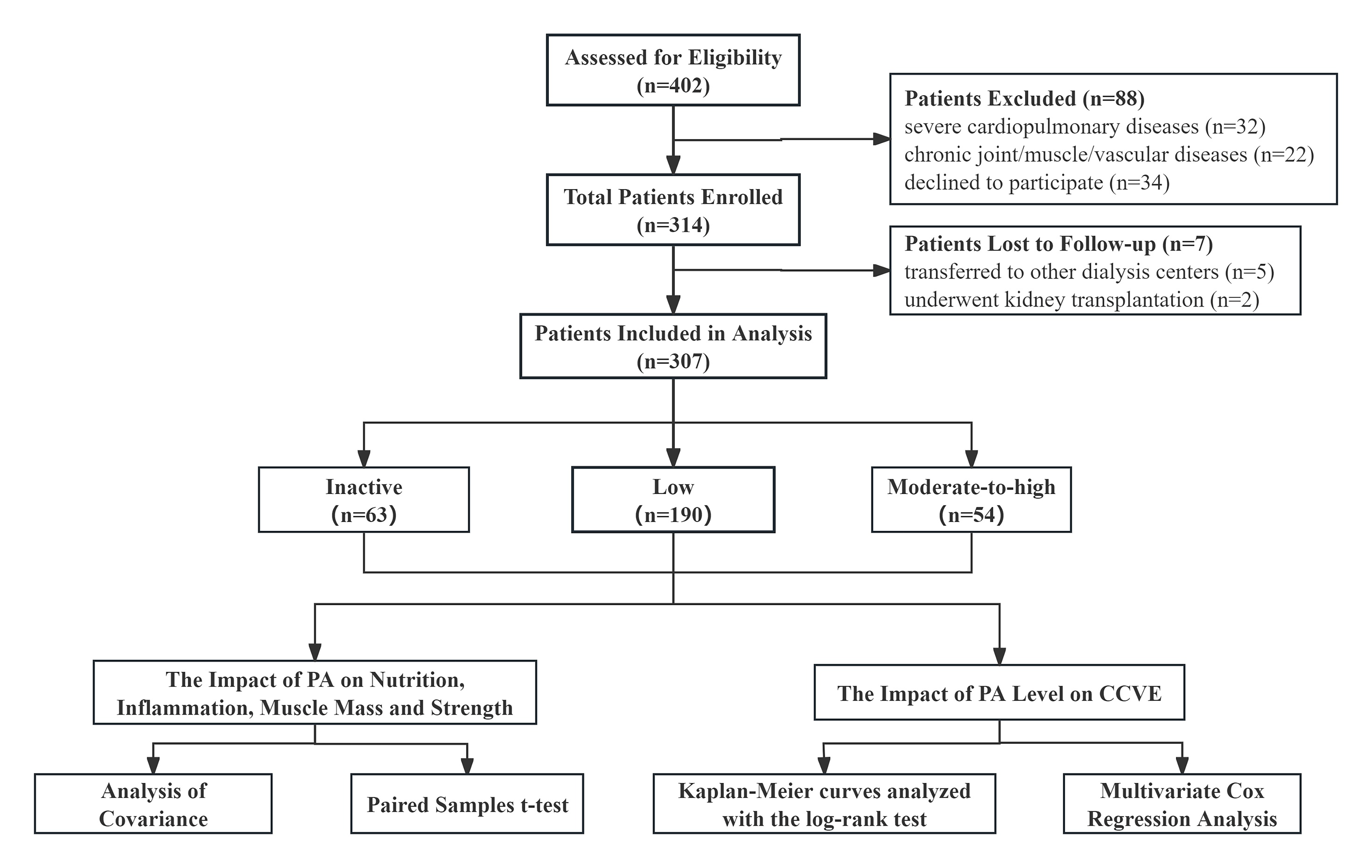

Supplement: Supplementary Figure S1.tif [file IRNF_A_2598982_SM9361.tif]
