# Supplementary material for: Effect of exercise on nutrition, inflammation, muscle health and cardio-cerebrovascular events in maintenance hemodialysis patients: a real-world prospective cohort study
Source: Ren Fail. 2025 Dec 18;47(1):2598982. doi: 10.1080/0886022X.2025.2598982 (PMC12720627; doi:10.1080/0886022X.2025.2598982)
Supplement: Supplementary Table S1.docx [file IRNF_A_2598982_SM9360.docx]

**Supplementary Table S1.** ANCOVA-adjusted comparison of physiological indicator differences between physical activity groups.

| Item | Differences between means (95% CI) compared with inactive group | |
| --- | --- | --- |
|  | Low | Moderate-to-high |
| MAC (cm) | 0.233 (-0.058~0.523) | 0.228 (-0.156~0.612) |
| MAMC (cm) | 0.396^a^ (0.087~0.706) | 0.664^b^ (0.258~1.070) |
| CC (cm) | 0.362^b^ (0.116~0.609) | 0.600^b^ (0.272~0.929) |
| HGS (kg) | 0.444 (-0.125~1.014) | 0.386 (-0.381~1.153) |
| Weighted HGS | 0.007 (-0.003~0.017) | 0.010 (-0.004~0.024) |
| Ishii score | -3.160^a^ (-5.679~-0.640) | -4.897^b^ (-8.302~-1.492) |
| MIS score | -0.765^b^ (-1.242~-0.289) | -1.109^b^ (-1.741~-0.476) |
| PAB (g/l) | 12.132 (-0.378~24.641) | 16.542 (-0.101~33.185) |
| Alb (g/l) | 0.553 (-0.347~1.453) | 0.666 (-0.530~1.862) |
| TRF (g/l) | 0.014 (-0.085~0.113) | 0.106 (-0.026~0.238) |
| LnCRP (mg/l) | -0.215 (-0.435~0.004) | -0.052 (-0.343~0.238) |
| Cr (umol/l) | 3.915 (-38.664~46.495) | -6.911 (-62.963~49.140) |

**Note:** ^a^ *P* < 0.05, ^b^ *P*＜0.01. Between group change by ANCOVA adjusted for baseline value, age, gender, dialysis duration, BMI, comorbidities (diabetes mellitus, CCVD), day protein intake.

**Abbreviation:** MAC: mid-arm circumference; MAMC: mid-arm muscle circumference; CC: calf circumference: HGS: handgrip strength; MIS score: malnutrition inflammation score; PAB: prealbumin; Alb: albumin; TRF: transferrin; LnCRP: natural logarithm of C-reactive protein; Cr: creatinine.
