# Supplementary material for: Effect of exercise on nutrition, inflammation, muscle health and cardio-cerebrovascular events in maintenance hemodialysis patients: a real-world prospective cohort study
Source: Ren Fail. 2025 Dec 18;47(1):2598982. doi: 10.1080/0886022X.2025.2598982 (PMC12720627; doi:10.1080/0886022X.2025.2598982)
Supplement: Supplementary Table S3.docx [file IRNF_A_2598982_SM9359.docx]

**Supplementary Table S3.** Variable Selection Process Using the Change-in-Estimate Criterion (10% Threshold).

| Characteristics | HR after removal (Low) | HR after removal (Moderate-to-high) | Retained |
| --- | --- | --- | --- |
| Male | 0.454 (0.250 ~ 0.824) | 0.224 (0.063 ~ 0.799) | No |
| Age | 0.424 (0.239 ~ 0.753) * | 0.175 (0.052 ~ 0.594) * | Yes |
| Dialysis duration | 0.462 (0.253 ~ 0.843) | 0.225 (0.063 ~ 0.801) | No |
| Smoking | 0.482 (0.268 ~ 0.867) | 0.235 (0.066 ~ 0.834) | No |
| With diabetes mellitus | 0.460 (0.258 ~ 0.822) | 0.224 (0.065 ~ 0.753) | No |
| With CCVD | 0.468 (0.261 ~ 0.837) | 0.224 (0.066 ~ 0.767) | No |
| Malnourished | 0.469 (0.256 ~ 0.859) | 0.230 (0.064 ~ 0.827) | No |
| RASIs user | 0.464 (0.260 ~ 0.826) | 0.222 (0.065 ~ 0.759) | No |
| Antiplatelets user | 0.488 (0.262 ~ 0.911) | 0.238 (0.066 ~ 0.860) | No |
| HGS | 0.484 (0.269 ~ 0.871) | 0.242 (0.069 ~ 0.852) | No |
| Weighted HGS | 0.482 (0.269 ~ 0.865) | 0.238 (0.069 ~ 0.819) | No |
| Ishii score | 0.473 (0.258 ~ 0.865) | 0.236 (0.066 ~ 0.847) | No |
| PAB | 0.453 (0.255 ~ 0.807) | 0.202 (0.059 ~ 0.689) * | Yes |
| Alb | 0.463 (0.256 ~ 0.837) | 0.224 (0.063 ~ 0.795) | No |
| CRP | 0.496 (0.264 ~ 0.929) | 0.239 (0.066 ~ 0.863) | No |
| TC | 0.452 (0.254 ~ 0.804) | 0.200 (0.059 ~ 0.680) * | Yes |
| LDL-C | 0.452 (0.254 ~ 0.804) | 0.209 (0.061 ~ 0.713) * | Yes |

**Note:** Initial HR (Low): 0.495 (0.264 ~ 0.928); Initial HR (Moderate-to-high): 0.239 (0.066 ~ 0.862)

* Indicates a change≥10% compared to the initial HR.

**Abbreviation:** CCVD: cardio-cerebrovascular disease; RASIs: renin-angiotensin system inhibitors; HGS: handgrip strength; PAB: prealbumin; Alb: albumin; CRP: C-reactive protein; TC: total cholesterol; LDL-C: low-density lipoprotein cholesterol.
